# Thyroid Hormone Hyposensitivity: From Genotype to Phenotype and Back

**DOI:** 10.3389/fendo.2019.00912

**Published:** 2020-01-24

**Authors:** Giuditta Rurale, Emery Di Cicco, Monica Dentice, Domenico Salvatore, Luca Persani, Federica Marelli, Cristina Luongo

**Affiliations:** ^1^Division of Endocrine and Metabolic Diseases, IRCCS Istituto Auxologico Italiano, Milan, Italy; ^2^Department of Clinical Medicine & Surgery, University of Naples Federico II, Naples, Italy; ^3^Department of Public Health, University of Naples Federico II, Naples, Italy; ^4^Department of Clinical Sciences and Community Health, University of Milan, Milan, Italy

**Keywords:** thyroid hormones, thyroid hormone hyposensitivity, thyroid hormone metabolism defects, thyroid hormone cell membrane transport defects, thyroid hormones action defects

## Abstract

Thyroid hormone action defects (THADs) have been classically considered conditions of impaired sensitivity to thyroid hormone (TH). They were originally referring to alterations in TH receptor genes (*THRA* and *THRB*), but the discovery of genetic mutations and polymorphisms causing alterations in cell membrane transport (e.g., *MCT8*) and metabolism (e.g., *SECISBP2, DIO2*) led recently to a new and broader definition of TH hyposensitivity (THH), including not only THADs but all defects that could interfere with the activity of TH. Due to the different functions and tissue-specific expression of these genes, affected patients exhibit highly variable phenotypes. Some of them are characterized by a tissue hypothyroidism or well-recognizable alterations in the thyroid function tests (TFTs), whereas others display a combination of hypo- and hyperthyroid manifestations with normal or only subtle biochemical defects. The huge effort of basic research has greatly aided the comprehension of the molecular mechanisms underlying THADs, dissecting the morphological and functional alterations on target tissues, and defining the related-changes in the biochemical profile. In this review, we describe different pictures in which a specific alteration in the TFTs (TSH, T4, and T3 levels) is caused by defects in a specific gene. Altogether these findings can help clinicians to early recognize and diagnose THH and to perform a more precise genetic screening and therapeutic intervention. On the other hand, the identification of new genetic variants will allow the generation of cell-based and animal models to give novel insight into thyroid physiology and establish new therapeutic interventions.

## Background

Thyroid hormones (TH, tetraiodothyronine T4 and triiodothyronine T3) are essential for proper embryonic development and postnatal regulation of growth and function of several organs. TH deficiency or excess during embryonic development results in profound and permanent defects, especially on the nervous system (1).

Circulating levels of TH are maintained within the reference range by feedback mechanisms operated by the Hypothalamic-Pituitary-Thyroid (HPT) axis. At targeted tissues, an adequate TH activity depends on the capability and availability of TH transporters (including but not limited to MCT8, MCT10, OATP1C1), deiodinases (DIO1, 2, and 3), and nuclear TH receptors (TRα1, TRβ1, and TRβ2) (2).

In humans, genetic defects in TH signaling lead to the onset of TH cell membrane transport defects (THCMTD), TH metabolism defects (THMD), or TH action defects (THAD), and are globally referred as TH hyposensitivity (THH) (3, 4).

Mutations of each gene are associated with characteristic alterations of thyroid function tests (TFTs). Nevertheless, some polymorphisms affecting TH transporter and deiodinase functions do not frankly alter TH profile, but a deeper investigation demonstrated that even slight modifications of deiodinase activity could have an impact on the treatment strategies for hypothyroidism (5).

In clinical practice, THH conditions are often difficult to recognize and treat. In fact, the TH profile in THH is not typical and readily recognizable as it happens in classic hyper- or hypothyroidism by routine TFTs. In addition, the clinical manifestations are not straightforward and represent the peculiar associations of peripheral tissue hyper- or hypothyroidism.

During the last two decades, the close collaboration between clinicians and basic researchers has been crucial to better understand the mechanism controlling intracellular TH activity, identify new genetic mutations and polymorphisms, and define the associated phenotypes. Importantly, the generation of specific animal models that mimic human THH conditions helped to unravel the pathophysiological mechanisms underlying these complex and variable phenotypes (6–9).

In this review, we summarize different clinical pictures in which a particular alteration of TH profile correlates with mutations or polymorphisms in a specific gene involved in TH transport (*MCT8*), TH metabolism (*SBP2, DIO2*), and TH activity (*THRA* and *THRB*).

## Normal/High TSH, Low FT4, High FT3: *MCT8* Mutations

The transmembrane passage of TH in cells is mediated by specific TH transporters (10). Among them, MCT8 facilitates the uptake and efflux of TH across the plasma membrane. In humans, inactivating mutations of *MCT8* cause the Allan-Herndon-Dudley syndrome, characterized by high serum T3, low T4, and slightly elevated TSH and neuromuscular defects (11). The phenotype can be more or less severe due to residual MCT8 activity. In fact, different kinds of mutations (e.g., substitution, deletion, insertion) variably affect the expression and activity of MCT8 (12). Moreover, the compensation operated by alternative TH transporters results in different alterations at tissue levels when *MCT8* is absent. In fact, some tissues appear normal or even hyperthyroid, whereas tissues that exclusively express *MCT8* present intracellular hypothyroidism (13, 14). Mct8 knockout mice (KO), Mct8/Oatpc1 and Mct8/Dio2 double KO mice provide useful information to understand the mechanism determining these alterations (15–17). The absence of MCT8 determines an increase in D1 and D2 activity that is responsible for the high levels of T3 and low T4 in serum. Moreover, the altered secretion of T3/T4 ratio by thyroid follicles described in Mct8 KO mice may also contribute to explain the low T4 serum content (16). Muscles isolated from Mct8 KO mice are hyperthyroid and showed impaired muscle regeneration, while Mct8/Oatpc1-depleted brains are hypothyroid (18). Recently, pluripotent stem cells (iPSCs) induced from MCT8-deficient patient can be efficiently differentiated into neural cells, and although TH transport is reduced, the TH transcription signature was normal (19). The authors demonstrated that the neurological phenotype is more related to the absence of TH transport throughout the blood-brain barrier than to an intrinsic deficit of MCT8 of the differentiated neurons. In light of these observations, the systemic thyroid status of MCT8-deficient patients cannot be classified as a generalized classical hypothyroidism or hyperthyroidism. This represents an important therapeutic challenge. Treatment with LT4 increased brain TH content but exacerbated the hypermetabolic state due to the increased D1 activity and then T3 production. Concurrently L-T4 and propylthiouracil (D1-inhibitor) administration normalized T4 without affecting T3 but failed to improve the neuromuscular phenotype (20). Lately, thyromimetic drugs as DITPA, Triac, and Tetrac have been proposed as treatment (21–23). In particular, Triac has been shown very effective in promoting neuronal differentiation when administered to Mct8 KO mice during the first postnatal week (24).

## Normal/High TSH, High FT4, Normal/Low FT3: *SBP2* Mutations

The characteristic thyroid signature of patients with biallelic inactivation of the *SBP2* gene is high T4, low/normal T3, and slightly elevated/normal levels of TSH. The *SBP2* gene codifies for a SECIS-binding protein involved in the incorporation of selenocysteine (Sec) into a family of selenoproteins (SPs) with diverse, essential, biological roles (25). The defective activity of SPs beside being involved in antioxidant defense and protein folding, affects TH metabolism since the deiodinases are selenocysteine-containing enzymes, thus resulting in a complex phenotype characterized by growth retardation, muscular dystrophy, intellectual disabilities, skin photosensitivity, hearing loss, insulin resistance, azoospermia, and aorthopathy (26). The observed phenotype is complex, but probably reflects three major pathogenic processes: (1) tissue-specific effects mediated by lack of a particular SP (e.g., the musculoskeletal phenotype caused by SEPN1 deficiency) (27); (2) consequences of more generalized tissue oxidative damage due to loss of antioxidant selenoenzymes with excess of cellular reactive oxygen species (e.g., aorthopathy) (26); (3) hypothyroid-related defects due to decreased activity of DIO2 and thus reduced peripheral T4-to-T3 conversion (e.g., growth delay, intellectual disability, and hearing loss) (28). However, whether and how these three mechanisms interplay in different tissues contributing to the SBP2 phenotype remain unknown. Several treatments (Se supplementation and T3 replacement) have been attempted to improve height and normalize TH levels, but only T3 treatment provided some beneficial effects (normal T3 levels, improved linear growth and neurodevelopment) (26, 29). Inducible, hepatocyte and neuron-specific Sbp2-deficient mouse models have not been reported to fully recapitulate SBP2 conditions, and constitutive Sbp2 KO mice die during embryonic life (30–32). Thus, additional model organisms are required to fully assess the pathophysiology responsible for thyroid phenotype and associated manifestations.

## TSH, FT3, And FT4 Within the Reference Range: *DIO2* Polymorphism

Deiodinases are essential to determine the intracellular concentration of THs. The expression of these three enzymes (D1, D2, and D3) is tissue and time dependent (33). Mice models demonstrated that life without deiodinases is allowed, but at the expense of alteration of sensory organs, metabolism, skeletal development, tissue regeneration, and HPT-axis regulation (34). Until today, mutations in deiodinases have never been reported in human conditions; we cannot discern whether this is due to relatively small effects of these mutations or to an incompatibility with life.

In patients, even slight alterations in TH levels have critical consequences on heart rate and bone mineral density; for this reason, the identification of different deiodinase polymorphisms affecting TH homeostasis is considered a topic of potential interest (5, 35, 36). Among them, the DIO2 Thr92Ala polymorphism has been associated with insulin resistance, obesity, hypertension, and alteration of hypothalamic-pituitary-thyroid axis (37). Notably, while individuals with the Thr92Ala polymorphism do not show an altered thyroid profile, athyreotic subjects carrying the Ala allele were associated with lower plasma fT3 level compared to wild type subjects, suggesting a decreased capability of peripheral T4 to T3 conversion due to a reduced DIO2 activity (5). In this study, the authors confirmed the partial D2 enzymatic deficiency by analyzing T4 sensitivity in pituitary and muscle cells. T4 was less effective in reducing TSH mRNA levels and in inducing apoptosis, respectively in pituitary primary cell culture and muscle cells transfected with the DIO2 Thr92Ala than in those transfected with the WT allele. The generation of a transgenic mouse carrying the DIO2 Thr92Ala polymorphism corroborated these findings. This mouse model showed hypothyroidism in distinct brain areas associated with memory, and behavior issues that improved after T3 administration were aggravated by primary hypothyroidism and responded only partially to LT4 treatment (38).

## Normal TSH, Low/Normal FT4, High/Normal FT3: *THRA* Mutations

Borderline variations in TFTs are frequently observed in patients with Resistance to Thyroid Hormone alpha (RTH α), a rare multisystem disorder caused by heterozygous mutations on the *THRA* gene (9). Two major receptor isoforms are produced from the *THRA* loci, TRα1 and TRα2, which differ in their carboxy terminus. Although TRα2 is incapable of binding TH, it is considered a modulator of TH action, antagonizing TRα1 activity (1). TRα1 is mainly expressed in the cerebral cortex, thyroid gland, heart, lung, gastrointestinal tract, liver, kidney, and ovary (39). RTHα patients present a complex and multisystem phenotype characterized by tissue-selective features of hypothyroidism. Based on the reports of nearly 30 patients with RTHα, the clinical phenotype includes, at varying degrees, growth restriction, impaired bone ossification and malformations, intellectual disability, bradycardia, and chronic constipation. A distinct facial dysmorphic phenotype consists of macrocephaly, broad facies, hypertelorism, flattened nose, prominent tongue, and thick lips. Some patients suffer from severe intellectual disability, epilepsy or autism-spectrum disorders, while others manifest only dyspraxia, ataxic gait, and dysarthria. Decreased basal metabolic rate, normochromic normocytic anemia, and elevated levels of creatine kinase (CK) and SHBG are also frequently observed in affected cases (9, 40–42).

RTHα patients typically present low FT4/FT3 ratio with low/normal FT4, high/normal T3, low serum rT3, and normal serum TSH levels (9). Mouse and zebrafish models of RTHα revealed that increased expression of hepatic DIO1 or hypothalamic DIO2 as well as reduced levels of DIO3 may be involved in generating this serum TH profile (43–46).

Because TH levels usually remain within the reference range, RTHα patients are not necessarily referred to endocrinologist, and it is likely that the disease frequently remains unrecognized by clinicians. In fact, only 21 heterozygous variants in *THRA* have been described in RTHα patients so far, but the latest release of the ExAC database (http://exac.broadinstitute.org) contains 68 *THRA* missense or frameshift mutations carried by anonymous patients, which are predicted to alter TRα1 function. Moreover, depending on the mutation, the clinical phenotype may vary among patients, ranging from severe to mild or minimal symptoms (41). All of known TRα mutations alter the ligand-binding domain (LBD) and include missense substitutions, deletions, and frameshift/premature stop codon, with dominant-negative (DN) properties. Functional *in vitro* and *in vivo* evaluations demonstrated that the missense substitution maintained a partial T3-binding activity, which can be normalized at high T3 doses, whereas the premature truncated variants showed an irreversible inability to recruit the coactivators acting as a constitutive transcriptional repressor (41, 45, 47, 48).

Thus, reduced FT4/FT3 ratio and increased SHBG and CK levels represent helpful serum parameters for diagnosis of RTHα. Moreover, a genetic screening of *THRA* should be considered in children with decreased growth rate, facial dysmorphic features, and delayed psychomotor development or in adults with unexplained constipation, megacolon, and bradycardia (49, 50). Very recently, characteristic metabolomic fingerprints in biofluids have been described in mice carrying human-like mutations (51). In particular, decreased levels of N-acetylglucosamine and choline/phosphocholine in urine or reduced levels of unsaturated lipids in serum might be used as additional biomarkers for the diagnosis of RTHα, likely discriminating patients with similar TH profiles (e.g., congenital hypothyroidism).

## Unsuppressed TSH, High FT4 and FT3: *THRB* Mutations

Elevated serum TH concentrations accompanied by non-suppressed TSH levels (inappropriate secretion of TSH: IST) are the hallmark of RTHβ, a rare disorder due to heterozygous mutations in the *THRB* gene causing a decreased response to TH action in the peripheral tissue (52). Two different TRβ (TRβ1 and TRβ2) isoforms are generated by alternate promoters on the *THRB* gene. TRβ1 is predominantly expressed in the brain, liver, and kidney, whereas TRβ2 is found in the pituitary, retina, and cochlea (39).

RTHβ is often diagnosed at childbirth during the screening for congenital thyroid dysfunction with abnormal level of T4 and TSH due to the impairment of the TRβ2-dependent regulation of the HPT-axis. As a consequence of the hyperstimulation of the thyroid gland, goiter is frequently present in patients with RTHβ (53, 54).

Since TRβ isoforms are expressed in few tissues, the RTHβ patients exhibited tissue-specific hypothyroid-related defects such as hepatic steatosis, dyslipidemia, and impaired hearing and color vision, together with thyrotoxic manifestations including attention-deficit hyperactivity disorder (ADHD), anxiety, tachycardia, and low bone density (53–55). RTHβ can appear in a sporadic form, but most commonly it is a familial syndrome with autosomal dominant inheritance. The clinical manifestations are variable between families with the same mutation and also between members of the same family with identical mutations. Differences in the functional properties of TRβ variants or other genetic and epigenetic modifications may influence the expression and the penetrance of the mutant receptor thus explaining the phenotypic variability of RTHβ. Additionally, high circulating TH levels can compensate the tissue resistance, and patients may appear euthyroid (55–60).

However, in a growing number of individuals, RTHβ occurs in the absence of mutations in the TR genes, likely due to defects in one of cofactors involved in TH signaling (61–63). Polymorphisms in TR-target genes have been associated with alterations in TFTs and may also account for the non-RTH patient's phenotype (64).

Over 160 different mutations in *THRB* have been identified so far, most of them localized to three hotspots within the LBD of the β receptors, which inhibit in a DN manner the transcriptional activity of TRs (2). As described for the RTHα, the type of the mutation correlates with the severity of the clinical phenotype and with the potential reversion of the DN activity by high T3 administration *in vitro* and *in vivo* (44, 65–67). The genetic screening of *THRB* should be considered in patients with normal/reduced serum SHBG levels in conjunction with high TH and unsuppressed TSH. Moreover, TH effects on neuromuscular and hepatic systems can also be addressed by the measurement of additional metabolic markers (e.g., SGOT, SGPT, cholesterol, triglycerides, ferritin, and CPK). A careful neurological examination looking for signs of hypothyroidism should be done to confirm the diagnosis of RTHβ.

## Conclusion

The identification of the physiological functions of genes involved in transport, metabolism, and action of TH in different tissues has been essential for a proper evaluation of the clinical manifestation of TH dysfunction. In particular, the advent of genetic manipulation techniques endorsed the generation of vertebrate knock-out and knock-in models that reproduce human mutations, providing a novel approach to study their functions in normal and pathological settings. Studies in vertebrate models with targeted gene manipulations allow uncovering the molecular mechanisms underlying THH defects and sometimes preceded the identification of the correspondent human genetic mutations (summarized in Table 1). For instance, murine TRα gene deletions were produced 15 years before the identification of the first *THRA* mutation (78, 94). Differently, the characterization of the phenotypes of DIOs-deficient mouse models was not accompanied by the identification of human genetic mutations so far (72, 73, 76).

**Table 1:** Animal models of THH.

**Gene**	**Species**	**Genotypes**	**Phenotypes**	**References**
*MCT8*	Mouse	Mct8^−/0^, Mct8^−/y^	Altered TH profile, tachycardia, increased metabolic rate, muscle wasting and cholesterol levels. No obvious neurological phenotypes (exhibited by Mct8^−/0^/Oatp1c1^−/−^ or Mct8^−/0^/Dio2^−/−^ mice)	(15, 16, 18, 68, 69)
	Danio rerio	Transient mct8 KD, stable mct8^−/−^	Neurological and behavioral alterations	(70, 71)
*SBP2*	Mouse	Sbp2^+/0^, hepatic-Sbp2^0/0^, neuron-Sbp2^0/0^	Mild to profound growth abnormalities, reduced expression of SPs, locomotor disabilities, severe impairment in neuron development	(30–32)
*DIO2*	Mouse	Dio2^−/−^	Impaired thermogenesis, increased T4 and TSH levels, defective myogenesis and muscle regeneration, hearing loss	(72–75)
	Mouse	Dio2^Thr92Ala^	Hypothyroidism in some brain areas, memory, and behavioral alterations	(38)
	Danio rerio	dio2^−/−^	Impaired TH metabolism (low T3 levels), hypothyroidism, growth retardation, locomotor, and fertility defects	(76, 77)
*THRA*	Mouse	TRα^−/−^, TRα1^−/−^, TRα1^0/0^, TRα1^7/7^, TRα1^PV/PV^, TRα1^AMI/+^, TRα1^R348C/R348C^, TRα1^E395fs401X/+^, TRα1^E395fs406X/+^, TRα1^395fs485X/+^, TRα1^K389fs479X/+^, TRα1^395fs485X/+^	Mild to profound hypothyroidism, growth retardation, delayed ossification, constipation, bradycardia, mild neurodevelopmental abnormalities, changes in metabolic profiling, reduced body temperature, anemia	(51, 78–84)
	Danio rerio	*thraa*MO, transient KI of hTRα1 mutants	Altered TH metabolism, growth retardation, delayed cartilages formation, cardiovascular defects, and bradycardia, anemia	(44, 45)
*THRB*	Mouse	TRβ^−/−^, TRβ^Tm1df/Tm1Df^, TRβ^Tm2df/Tm2Df^, TRβ2^−/−^, TRβ^PV/PV^, TRβ^D337T/D337T^, TRβ^R429Q/R429Q^, TRβ^G345R/G345R^, TRβ^PV/PV^,	Resistance to TH, inappropriate TSH secretion, goiter, short stature, impaired cholesterol metabolism, impaired photoreceptor differentiation and auditory functions, hepatic steatosis, tachycardia	(85–92)
	Daniorerio	Transient KD of TRβ1/TRβ2 or TRβ2	Hypothalamic and pituitary resistance to TH, thyroid enlargement, growth retardation, defective differentiation of photoreceptors and impaired otic vesicle development	(44, 93)

**Figure 1:**
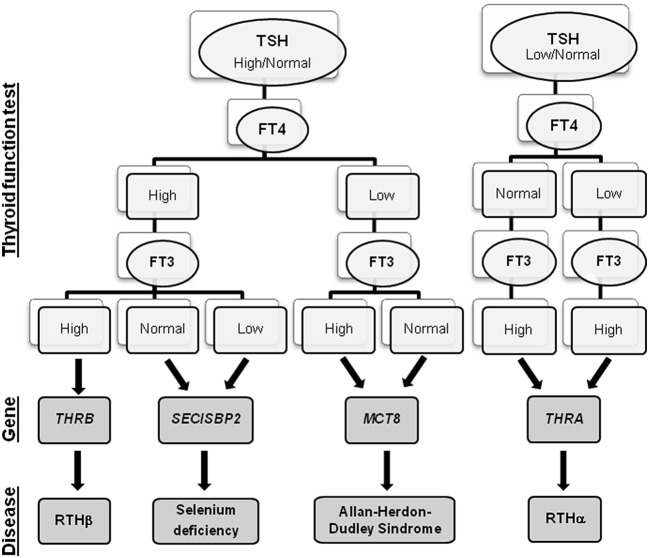
Flowchart of THH diagnosis. For each THH disease, the associated gene and the alterations in the thyroid function tests are reported.

The impact of neonatal screening programs for congenital hypothyroidism facilitates early diagnosis of such a devastating disorder, resulting in immediate treatment. The possibility to measure TSH and TH in dried spots has huge potential since it facilitates the identification of the different thyroid signatures characterizing THH in the first week of life. What complicates this only apparent simple picture is the high variability of biochemical and clinical phenotypes among patients with similar or even identical genetic mutations. In fact, while some patients present only subtle TH alterations and mild clinical features, others exhibit abnormal TFTs and severe clinical abnormalities. So, changes of metabolic markers together with the observation of certain clinical manifestations are required to recognize the different THH conditions. Moreover, since similar alterations in TFT can be found in patients with different thyroid diseases (e.g., congenital hypothyroidism), these additional indicators should be taken into account during the differential diagnosis. The biochemical signatures associated with each gene mutations are summarized in Figure 1.

In conclusion, the combined effort of clinical and basic research is the unmet need to improve the diagnosis of the different forms of THH. The identification of new patients and mutations can deeply improve our understanding of the physiologic role of the genes involved in THH but will also provide further insights into the molecular mechanisms underlying each phenotype.

Such information may also facilitate the design of new drugs that can be tested in animal models, as a prelude to subsequently undertaking proof-of-concept clinical trials in patients.

## Author Contributions

GR, EC, FM, and CL wrote the manuscript. MD, DS, and LP supervised and finalized the manuscript.

### Conflict of Interest

The authors declare that the research was conducted in the absence of any commercial or financial relationships that could be construed as a potential conflict of interest.
